# Supplementary material for: Mono- and multidomain defense toxins of the RelE/ParE superfamily
Source: mBio. 2025 Feb 25;16(4):e00258-25. doi: 10.1128/mbio.00258-25 (PMC11980606; doi:10.1128/mbio.00258-25)
Supplement: Supplemental Legends — Legends to supplemental figures and tables. [file mbio.00258-25-s0008.docx]

**Legends to Supplementary Figures and Tables**

**Figure S1. Clustering analysis of PF_ParE and PF_ParE-like monodomain toxins.**

Clustering analysis of 3,605 toxin sequences belonging to the PF_ParE (black dots) and PF_ParE-like (red dots) families, visualized in two dimensions using CLANS. Experimentally validated toxins with confirmed cellular targets are shown as green dots. Five distinct subclusters of PF_ParE toxins are highlighted with blue ellipses. The ribosome-dependent mRNases RelE of *E. coli* K-12 and *Nostoc* belonging to Subcluster #4 are indicated with arrows.

**Figure S2. Structures of RelE, YiaG and RelE-YiaG fusion proteins.**

1. AF2 model of RelE from *E. coli* VREC0535 (SQP90372.1). The model reveals an extended, unstructured N-terminal domain typical of RelE toxins in this module. The structured BECR-fold is located in the C-terminal region.
2. Structures of YiaG. Upper panel: AF2 model of YiaG (SQP90371.1) from the *relE yiaG* TA module of *E. coli* VREC0535. The HTH domain is highlighted in grey, while the remaining regions are colored red. Lower panel: Superimposition of the HTH domains of YiaG (*E. coli* VREC0535) and HigA-2 (*V. cholerae*, AAF96373.1) shows structural similarity, with an RMSD of 0.833 Å over 56 amino acid pairs.
3. Quaternary structure of the RelE₂YiaG₂ complex. The modeled heterotetramer of RelE₂YiaG₂ from *E. coli* VREC0535, with RelE subunits shown in blue (as in panel A) and YiaG subunits in red and grey (as in panel B). Structure quality assessment metrics calculated by ModFPOLDdock are as follows: plDDT = 0.831; pTM = 0.502; Assembly quality = 0.7437; Interface quality = 0.6368.
4. AF2 model of the RelE-YiaG fusion protein from *S. enterica* subsp. *enterica* (SUG72942.1). The BECR-fold domain (toxin) is shown in blue, the YiaG antitoxin domain in red, and the C-terminal HTH domain in grey.
5. Structural superimposition of the YiaG antitoxin domain of *E. coli* VREC0535 (SQP90371.1) and the C-terminal antitoxin domain of the *S. enterica* RelE-YiaG fusion protein, yielding an RMSD of 0.595 Å over 63 pruned atom pairs. The N-terminal helices of both antitoxin domains align closely, supporting structural conservation.

**Figure S3. YefM-YoeB and RHH-YoeB fusion proteins.**

1. Schematic representation of gene arrangements in *yefM-yoeB* toxin-antitoxin (TA) systems. Two-gene loci encode separate YefM antitoxin and YoeB toxin proteins, while monogene TA loci encode fused proteins containing either a YefM DNA-binding domain or a RHH domain linked to the BECR-fold YoeB toxin domain.
2. Model of the monomeric YefM-YoeB fusion protein from *M. shinjukuense* (BBX74620.1; 179 aa) generated by AF2. The YefM domain is shown in red and the BECR-fold YoeB domain in cyan. The interdomain region is unstructured. Model quality is high (plDDT > 90 for most residues).
3. Model of the dimeric YefM-YoeB fusion protein from *M. shinjukuense* (BBX74620.1; 179 aa), generated using MultiFold. Structural evaluation using ModFOLDdock indicates strong assembly and interface quality (plDDT = 0.911, pTM = 0.839, Assembly Quality = 0.8693, Interface Quality = 0.8454).
4. Overlay of the DNA-binding domains of the YefM-YoeB fusion protein from *M. shinjukuense* and the YefM domain of the Phd protein from phage P1 (blue). RMSD = 0.696 Å over 41 pruned atom pairs, indicating close structural similarity. The YefM domain from *M. shinjukuense* is shown in red.
5. AF2-generated model of an RHH-YoeB fusion protein (CDN32595.1; 233 aa) from *Mucinivorans hirudinis*. The RHH DNA-binding domain is shown in red, and the BECR-fold YoeB domain in cyan, illustrating the modular architecture of these TA systems.

**Figure S4. Superimposition of ATPases PtuA of *E. coli* and PtuY of *B. ovatus*.**

The cryo-EM structure of PtuA of *E. coli* ATCC 25922 (AIL15948.1; magenta) ([94](#_ENREF_94)) was superimposed on the AF2 model of PtuY of *B. ovatus* (KAB1323966.1). The superimposition yielded an RMSD between 99 N-terminal pruned atom pairs of 1.054 Å.

**Figure S5. Quaternary models of PtuY dimers and dimer-trimers of *E. coli* 115A and *B. ovatus*.**

1. Predicted dimer structure of PtuY from *E. coli* 115A (RQN67039.1), with surface interactions and quality metrics calculated using ModFOLDdock. Individual subunits are shown in distinct colors. Quality parameters: plDDT = 0.972, pTM = 0.958, Assembly quality = 0.9487, Interface quality = 0.9502. Interacting residues are shown in blue.
2. Predicted dimer structure of PtuY from *B. ovatus* (KAB1323966.1), colored by interface quality. Quality metrics: plDDT = 0.981, pTM = 0.968, Assembly quality = 0.944, Interface quality = 0.9488. Interacting residues are shown in blue.
3. Trimer-of-dimers model of PtuY from *E. coli* 115A, where each subunit is colored individually to highlight structural arrangement. Model was generated using MultiFold. Quality metrics: plDDT = 0.953, pTM = 0.849.
4. Trimer-of-dimers model of PtuY from *E. coli* 115A, highlighting interacting residues in blue to emphasize subunit interfaces. Interacting residues are shown in blue.
5. Trimer-of-dimers model of PtuY from *B. ovatus*, with each subunit individually colored. Model generated using MultiFold. Quality parameters: plDDT = 0.976, pTM = 0.965.
6. Hexameric assembly of PtuY from *B. ovatus*, where interacting residues are shown in blue, highlighting inter-subunit interfaces.

**Figure S6. Structural and functional characterization of a transmembrane BECR-fold toxin encoded by a toxin-antitoxin module**

1. Structural model of ToxM from *Xanthomonas perforans* (APP00616.1) generated by MultiFOLD and Membranefold. The model highlights an intracellular BECR-fold RNase domain (blue), the transmembrane (TM) domain (red), and an extracellular disordered region (pink). Domains are colored based on predicted cellular localization.
2. Same structural model as in panel A, now colored according to predicted quality of individual residues using plDDT scores. Blue shades indicate high-confidence regions (plDDT > 90).
3. Transmembrane topology prediction of ToxM using Membranefold. The plot indicates high-confidence localization of the TM domain (red) and the distribution of intracellular (inside) and extracellular (outside) regions.
4. Dimer model of ToxR (APP0061/.1), the antitoxin counterpart of ToxM, generated by AF2. ToxR monomers are shown in light and dark blue and are composed primarily of three helices. Quality assessment: plDDT = 0.783, pTM = 0.643, ipTM = 0.638.
5. Tetrameric complex model of ToxM's intracellular BECR-fold domains (red and purple) interacting with the ToxR dimer (dark and light blue). The structure was generated using MultiFOLD and evaluated by ModFOLDdock. Quality metrics: plDDT = 0.774, pTM = 0.681, Assembly Quality = 0.683, Interface Quality = 0.604. The N-terminal regions of ToxR interact with ToxM, wrapping around the toxin, suggesting a potential inhibitory mechanism.

**Legends to Supplementary Tables**

**Table S1. Thirteen families of BECR-fold monodomain toxins encoded by type II TA modules.** This table contains the primary data of the thirteen protein families containing BECR-fold toxins that are encoded by type II toxin-antitoxin (TA) modules and analysed in this work. Protein sequences were retrieved from the NCBI database (<https://www.ncbi.nlm.nih.gov/protein/>), UniProt database (<https://www.uniprot.org/>) or InterPro database (<https://www.ebi.ac.uk/interpro/>) and proportionally reduced to approximately 9,000 sequences in total for comprehensive analysis. The PF_ParE family was further subdivided into five distinct clusters based on clustering analysis (**Figure S2**), and these clusters are also included in the Table. Toxins highlighted in blue indicate their inclusion in the analysis presented in the main text. For relevant toxins, their cognate antitoxins are listed alongside. An asterisk (*) next to a toxin's PF_ID denotes that its cellular target has been experimentally verified. GB_ID: GenBank Identifier.

**Table S2. Multidomain BECR-fold toxins.** This table summarizes the details of four types of multidomain BECR-fold toxins, including the InterPro and GenBank identifiers, protein sequences, distances between toxin and antitoxin genes (in base pairs where a minus indicates size of gene overlap), genus and taxonomy. The four types of multidomain BECR-fold toxins are: Antitoxin – toxin gene fusions, toxin – antitoxin gene fusions, the novel *ptuXYZ* defence modules, TA loci encoding toxins with a transmembrane toxin.
